# Modulation of Oxidative Stress in Diabetic Retinopathy: Therapeutic Role of Natural Polyphenols

**DOI:** 10.3390/antiox14070875

**Published:** 2025-07-17

**Authors:** Verónica Gómez-Jiménez, Raquel Burggraaf-Sánchez de las Matas, Ángel Luis Ortega

**Affiliations:** 1 Department of Physiology, Faculty of Pharmacy, University of Valencia, Vicente Andrés Estellés Av. s/n, 46100 Burjassot, Spain; gojive@alumni.uv.es; 2 Ophthalmology Department, Vitreoretinal Unit, Hospital of Sagunto, Ramón y Cajal Av. s/n, 46520 Valencia, Spain; burggraaf_raq@gva.es

**Keywords:** diabetic retinopathy, oxidative stress, antioxidant therapy, nanotechnology, polyphenols, retinal neurovascular damage

## Abstract

Diabetic retinopathy (DR), a leading cause of blindness in working-age adults, arises from chronic hyperglycemia-induced oxidative stress, inflammation, and vascular dysfunction. Current therapies such as laser photocoagulation, intravitreal anti-vascular endothelial growth factor (VEGF) agents, and steroids target advanced stages but fail to prevent early neuronal and microvascular damage. Emerging evidence highlights oxidative stress as a key driver of DR pathogenesis, disrupting the blood-retinal barrier (BRB), promoting neurodegeneration and angiogenesis. Advances in imaging, particularly optical coherence tomography angiography (OCTA), enable earlier detection of neurodegeneration and microvascular changes, underscoring DR as a neurovascular disorder. Polyphenols, such as resveratrol, curcumin, and pterostilbene, exhibit multitarget antioxidant, anti-inflammatory, and anti-angiogenic effects, showing promise in preclinical and limited clinical studies. However, their low bioavailability limits therapeutic efficacy. Nanotechnology-based delivery systems enhance drug stability, tissue targeting, and sustained release, offering potential for early intervention. Future strategies should integrate antioxidant therapies and precision diagnostics to prevent early irreversible retinal damage in diabetic patients.

## 1. Introduction

Diabetes mellitus (DM), a chronic metabolic disease characterized by sustained hyperglycemia that damages multiple organ systems, is among the top ten causes of death worldwide [1,2]. Its prevalence continues to rise, with an estimated 46% increase projected by 2045, according to the International Diabetes Federation [3]. The mortality associated with DM, along with reduced quality of life and productivity, is largely driven by its microvascular and macrovascular complications [4]. Diabetic retinopathy (DR) is considered one of the most common microvascular consequences of DM. DR is a progressive, asymptomatic neurovascular complication that leads to irreversible retinal damage [5], which represents the leading cause of vision loss among working-age adults (20–65 years) and the fifth leading cause of blindness worldwide [6,7]. In its early stages, chronic hyperglycemia disrupts the integrity of the blood-retinal barrier (BRB), increasing vascular permeability and potentially leading to capillary occlusion, which characterizes non-proliferative diabetic retinopathy (NPDR). In advanced stages, retinal hypoxia induces pathological neovascularization, delineating the transition to proliferative diabetic retinopathy (PDR). Additionally, fluid accumulation in the macula can result in diabetic macular edema (DME), compromising central visual acuity [8,9,10].

Approximately one-third of individuals with DM will develop DR, with a prevalence reaching 77.3% in DM type 1 (DM1) and 25.1% in DM type 2 (DM2) [11,12]. Among DM2 patients, 6.99% of cases occur in pediatric populations [13]. Notably, DR is the only cause of blindness whose prevalence has steadily increased over the past four decades (1980–2018) [14]. Moreover, its prevalence is expected to continue rising in the absence of effective therapeutic strategies, with a growing burden in low- and middle-income regions [15,16].

DR remains predominantly asymptomatic in its early stages, delaying diagnosis and allowing progression to advanced stages with irreversible damage [17]. Throughout this process, oxidative stress induced by chronic hyperglycemia plays a crucial role in both vascular and neuronal deterioration of the retina [18,19,20,21]. In this review, our aim is to highlight the limitations of currently used conventional therapies and, based on the oxidative etiology of DR, to explore in depth the prevention of disease progression through the use of natural polyphenols.

## 2. Conventional Therapies and Therapeutic Effects

Visual impairment in patients with DR can be caused by the development of DME, which implies the presence of fluid in the macula (the central area of the retina) and can occur at any stage of the disease. Visual loss may also result from bleeding or tractional retinal detachments due to the presence of neovessels; in the context of PDR, this is the most advanced stage of DR. According to a 2021 meta-analysis, the prevalence of DME was estimated to be approximately 4.07% among the diabetic population, while vision-threatening DR affected about 6.17% of people in this category [7]. Most patients may remain visually asymptomatic for long periods of time if they do not develop any of the mentioned situations. For this reason, screening visits are recommended, generally involving periodic fundus photographs, which should be performed three to five years after the diagnosis of DM1 and from the time of diagnosis in DM2 [22,23].

**Figure 1:**
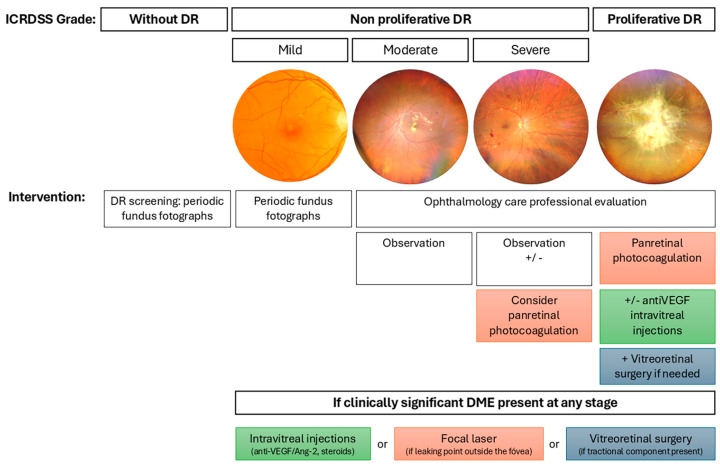
Management of DR according to severity based on the International Classification of Diabetic Retinopathy Severity Scale. The figure outlines the clinical approach to DR across its progressive stages: no apparent DR, NPDR (mild, moderate, severe), and PDR. Representative fundus photographs illustrate the retinal appearance at each stage. Recommended interventions are indicated for each level of severity, ranging from periodic fundus imaging and observation to panretinal photocoagulation, intravitreal injections (anti-VEGF, angiopoietin-2 (Ang-2) inhibitors, corticosteroids), and vitreoretinal surgery when necessary. Additionally, treatment options for clinically significant diabetic macular edema (DME) are specified, depending on the presence and anatomical characteristics of the edema.

In routine clinical practice, treatments applied according to the DR management guidelines are laser retinal photocoagulation, intravitreal injections, and vitreoretinal surgery. Figure 1 summarizes the indications for each treatment based on the degree of DR according to the International Classification of Diabetic Retinopathy Severity Scale [24]. These conventional therapies are used in cases of severe NPDR, PDR, or DME [22,23].

Laser photocoagulation is indicated in any case of PDR and in cases of severe NPDR where rapid progression to PDR is expected, when the following risk factors are present: pregnancy, contralateral PDR, non-compliance with appointments, poor glycemic control, large ischemic areas, or the presence of cataract-impeding laser application in the short or medium term. In these cases, a panretinal photocoagulation (PRP) is performed, applying laser to the entire peripheral retina, while sparing the macular area, which is responsible for visual acuity (VA) [22,23]. Conventional argon laser treatment causes thermal burns in the outer retina. This reduces the oxygen demands from the ischemic retina while allowing oxygen supply from the choriocapillaris to diffuse into the inner retina, thereby decreasing vascular endothelial growth factor (VEGF) production. This promotes the regression of existing neovascularization and prevents the formation of further erratic neovessels [25]. Side effects would include peripheral visual field loss [22,25]. New instruments or techniques with less aggressive parameters are currently used to minimize retinal tissue damage. These include Pattern Scanning Laser Photocoagulation, subthreshold diode micropulse photocoagulation, navigated laser photocoagulation, targeted retinal photocoagulation, photobiomodulation therapy, and photo-mediated ultrasound therapy. However, further randomized clinical trials are necessary to determine their applications and effectiveness in comparison to conventional argon laser strategy [25,26].

A second indication for laser is the presence of non-center-involving DME when a leaking microaneurysm is evident as the origin of the macular fluid, located at least 500 microns away from the fovea, the center of the macula. An impact is applied directly to the leaking point to occlude it by generating a scar [23,27].

Intravitreal injections are the gold-standard treatment for center-involved DME when VA is worse than 20/25 [28]. Currently available intravitreal drugs focus on reducing inflammatory cytokines, inhibiting VEGF and, more recently, the angiopoietin-2 (Ang-2). These drugs make it possible to stop the leaking of fluid within the macula, restoring VA. Table 1 shows the drugs approved by the Food and Drug Administration (FDA) and the European Medicines Agency (EMA) for the treatment of DME and the therapeutic targets on which they act [29]. Due to the high cost of these injections, biosimilar medicines have begun to be developed to reduce the economic burden of this pathology [30].

For anti-VEGF injections, there are several treatment regimens. In cases where close monitoring of the patient is possible, a pro re nata strategy can be used, which consists of administering a single intravitreal injection followed by monthly monitoring to assess whether the patient requires another injection. The current strain on health systems, with the inability to conduct exhaustive check-ups on all patients, led to the establishment of treat-and-extend regimens. These involve three to five initial monthly injection procedures, followed by new doses at longer intervals (extending to every 8 or 12 weeks, or, in the case of drugs more recently approved by the FDA/EMA, up to every 16 weeks for faricimab [31] or aflibercept (8 mg)) [22].

A meta-analysis evaluating different anti-VEGF agents in 2023 (including bevacizumab, ranibizumab, aflibercept (2 mg), brolucizumab, and faricimab) determined a mean number of injections ranging from seven to 12 within the first year of treatment. Every treatment improved VA at 24 months, although the difference when comparing anti-VEGF agents was unlikely to be significant [32]. Brolucizumab and faricimab demonstrated longer durability, requiring fewer injections. However, caution must be taken with brolucizumab, given the higher incidence of intraocular inflammation events; close monitoring is required to detect them [33,34]. The authors of the meta-analysis warned that the results of the included randomized clinical trials may differ from real-world settings, as treatment regimens may be less strict [32]. Indeed, a multicenter real-world data review from the Fight Retinal Blindness Study Group evaluating bevacizumab, ranibizumab, and aflibercept (2 mg) reported in 2024 a frequency of four to seven injections during the first 12 months of treatment [35].

Other uses for anti-VEGF injections include their administration less than five days prior to vitreoretinal surgery in order to reduce intra- and postoperative vitreous cavity bleeding [36]. Their application in cases of moderate to severe NPDR and PDR to eliminate or prevent neovascular formation is also being studied and does not meet with approval in this context worldwide. The Diabetic Retinopathy Clinical Research Network determined that patients with moderate to severe NPDR treated with prophylactic aflibercept (2 mg) for 2 years, and if needed between the second and the fourth year, presented statistically significant milder stages of DR after 4 years, but there were no benefits in terms of VA [37]. Due to the high cost of injections and the need for extensive monitoring, these treatments have not yet demonstrated their definite applicability in patients without center-involved DME. For PDR, the Diabetic Retinopathy Clinical Research Network reported that ranibizumab given monthly for 24 weeks, allowing for deferral from the sixteenth week if neovascularization regressed, was not inferior to PRP after 5 years of follow-up, with similar VA [38]. Nevertheless, treatment with ranibizumab between the fifth and the tenth year was only cost-effective for patients with center-involved DME [39]. The CLARITY study demonstrated that aflibercept was not inferior to PRP after 1 year, reducing complications in terms of DME, vitreous cavity bleeding, the need for vitrectomy, or visual loss [40]. However, anti-VEGF injections do not produce reperfusion of the retina [41], and stopping injections may significantly worsen the DR. It has been demonstrated that patients with PDR treated with anti-VEGF injections and then loss to follow-up present a higher incidence of tractional retinal detachment and iris neovascularization in comparison to PRP [42], meaning that the long-term application of anti-VEGF in PDR must be applied with caution.

In the case of intravitreal steroids, the biodegradable implant Ozurdex^®^ provides an intraocular duration of up to 6 months, while Iluvien^®^ is a non-biodegradable sustained-release drug delivery system that lasts for 36 months. These drugs are particularly indicated in cases where DME shows predictive inflammatory biomarkers on retinal imaging of the macula [43]. The treatment regimen for these implants begins with a single dose of Ozurdex^®^ to assess its effect and potential side effects, such as an increase in intraocular pressure. After 4 months, the need for another injection is evaluated. If recurrent DME responds to Ozurdex^®^, Iluvien^®^ is available as a long-term treatment [22].

When considering the cost of the intravitreal injections, including medication, procedure, and follow-up evaluation, a French study estimated that the average annual cost during the first year of treatment was €5782 for aflibercept (2 mg), €2779 for dexamethasone, and €5536 for ranibizumab [44]. These findings are consistent with a prior German systematic review, which reported 3-year costs of €17,540 for ranibizumab, €15,894 for aflibercept, €10,822 for the fluocinolone acetonide implant, and €12,363 for the dexamethasone implant [45]. In the United States, where off-label use of bevacizumab is more prevalent, and often required by insurance companies, the Diabetic Retinopathy Clinical Research Network reported 2-year costs of $13,929 for a bevacizumab-first strategy, compared to $26,504 for aflibercept monotherapy. Notably, 70% of patients initially treated with bevacizumab needed to switch to aflibercept by the end of the 2-year period [46]. Biosimilar products offer the potential for more affordable treatment options, typically reducing costs by approximately 30% compared to the reference drug, though they remain more expensive than off-label alternatives like bevacizumab [30].

The last conventional therapy that deserves to be mentioned is the pars plana vitrectomy, which is indicated in cases of PDR when there is a non-clearing vitreous hemorrhage (especially without prior PRP), in cases of tractional retinal detachment involving the macula, combined tractional-rhegmatogenous retinal detachment, or when a dense pre-macular subhyaloid hemorrhage is present [22]. A second scenario is the presence of DME influenced by vitreomacular traction, with the aim of reducing macular thickness [47].

As mentioned, the set of therapies currently used focuses on addressing the disease in advanced stages to prevent its progression to blindness [21]. Due to the inability to regenerate neural and vascular tissue appropriately, it would be more appropriate to prevent the tissue damage induced by diabetes from occurring in the first place. With this goal, efforts are made to raise awareness among patients about the importance of maintaining good glycemic control, but in many cases, it has little effect on the population. This situation leads us to consider other approaches that can protect retinal tissue, for which an in-depth study of the disease and the molecular causes that induce the damage is necessary.

## 3. Oxidative Stress and Classical Biochemical Mechanisms as Inducers of Diabetic Retinopathy

The human retina requires approximately 300% and 600% more oxygen than the cerebral cortex and cardiac muscle, respectively [48]. Due to this high demand, both the retina and its vascular system are particularly susceptible to oxidative stress [49]. Oxidative stress arises from an imbalance between the production of reactive oxygen species (ROS) and the body’s antioxidant capacity to neutralize them [18]. Through glycolysis, glucose is metabolized into pyruvate, which subsequently enters the mitochondria to produce adenosine triphosphate (ATP) via the respiratory chain [18,50]. Chronic hyperglycemia and the resultant metabolic overload trigger various pathological alterations that contribute to microvascular and neuronal damage through ischemic and hyperosmotic mechanisms, while also exacerbating oxidative stress [49,51].

Key metabolic pathways activated in DR include the polyol pathway [50], the hexosamine pathway [52], protein kinase C (PKC) signaling [53], and the accumulation of advanced glycation end products (AGEs) [54]. These pathways promote the activation of proliferative growth factors, such as VEGF, and exacerbate ROS overproduction, inflammation, cellular apoptosis, and BRB disruption, ultimately leading to vascular and neuronal damage in the retina [55,56]. In parallel, hyperglycemia compromises endothelial function [57] and disrupts vascular homeostasis, while extracellular matrix alterations and neuronal dysfunction further contribute to retinal deterioration [58]. Moreover, inflammation and immune responses, characterized by leukocyte adhesion, chemokine overexpression, and increased inflammatory mediators, play a key role in disease pathogenesis [59].

The polyol pathway converts excess glucose, which cannot be phosphorylated by hexokinase due to saturation, into sorbitol via aldose reductase, using nicotinamide adenine dinucleotide phosphate (NADPH) as a cofactor. Sorbitol is subsequently converted into fructose by sorbitol dehydrogenase with the aid of NAD^+^ [50,60]. Excessive activation of aldose reductase has been associated with damage to various retinal cell types, including endothelial cells [61], pericytes [62], ganglion cells [63], Müller glial cells [63], retinal pigment epithelial cells [63], photoreceptors, and amacrine cells [64]. The deleterious effects of the polyol pathway on the viability of multiple retinal cell types in DR arise from several mechanisms. First, the consumption of NADPH and NAD^+^ reduces glutathione (GSH) regeneration, weakening antioxidant defenses and promoting ROS accumulation, leading to oxidative stress [60]. Second, intracellular sorbitol accumulation, due to its low permeability across cell membranes, induces osmotic stress, increasing water retention and causing cellular damage [65,66]. Third, fructose produced in this pathway is converted into fructose-3-phosphate, which degrades into compounds that promote the formation of AGEs [67].

The hexosamine biosynthetic pathway is activated when excess fructose-6-phosphate, a glycolytic intermediate, is diverted from glycolysis [52]. This pathway contributes to DR through three main mechanisms: the inhibition of insulin/Protein Kinase B (Akt) signaling [68], promoting neuronal death; the enhancement of inflammation via increased O-GlcNAcylation of nuclear factor κ B (NF-κB) p65, leading to ganglion cell injury; and the induction of pericyte apoptosis through p53 modification, resulting in vascular dysfunction and capillary instability [69].

PKC activation is another key mechanism in DR progression. Hyperglycemia elevates levels of glyceraldehyde-3-phosphate (GA-3P), a glycolytic intermediate that enhances diacylglycerol production and activates PKC isoforms in the retina [53]. Diacylglycerol accumulation activates PKC-β, which alters enzymatic activities of nitric oxide synthase (NOS), as well as the expression of endothelin-1, and VEGF in endothelial cells, leading to vascular dysfunction [70,71]. Additionally, PKC-δ activation promotes pericyte loss by increasing oxidative stress and downregulating the platelet-derived growth factor survival signaling pathway [53].

Chronic hyperglycemia also promotes AGE formation and accumulation in the extracellular matrix and endothelial cells of retinal capillaries [54]. AGEs contribute to retinal damage by inducing aberrant cross-linking of extracellular matrix proteins, increasing vascular stiffness, and compromising structural and functional integrity [72]. Moreover, the interaction of AGEs with their receptor (RAGE) initiates intracellular signaling cascades that amplify the expression of pro-inflammatory cytokines, pro-angiogenic factors, endothelial dysfunction, pericyte apoptosis, and the breakdown of the inner BRB [54,72,73].

In addition, hyperglycemia-induced ROS production activates poly (ADP-ribose) polymerase, which inhibits glyceraldehyde-3-phosphate dehydrogenase (GAPDH) [74]. This inhibition facilitates further activation of the polyol pathway, enhances AGE formation via methylglyoxal, activates PKC and NF-κB, and increases flux through the hexosamine pathway [75]. Within this pathway, glutamine:fructose-6-phosphate amidotransferase regulates transforming growth factor β (TGF-β) expression and PKC activation in response to glucose [76]. Concurrently, excess glucose is converted into sorbitol, inducing osmotic stress before being metabolized to fructose [65,66]. The byproducts of this process, including fructose-3-phosphate and 3-deoxyglucosone, are potent glycation agents that further promote AGE formation. In turn, AGEs enhance oxidative stress and reinforce PKC activation, perpetuating the cycle of retinal damage in DR [77].

Beyond metabolic alterations, abnormal epigenetic modifications, NF-κB activation, decreased nuclear factor erythroid 2- related factor 2 (Nrf2) activity, and hyperglycemia-induced mitochondrial dysfunction contribute to ROS overproduction in DR [55]. Importantly, oxidative stress resulting from epigenetic changes can persist even after glycemic control is achieved, a phenomenon termed “metabolic memory” [78]. Among the relevant epigenetic alterations involved in DR, an increase in S-adenosylmethionine (SAM)-mediated DNA methylation has been observed in patients, particularly affecting the matrix metalloproteinase 9 (MMP-9) gene, which has been closely linked to the disease [79]. Additionally, acetylation of NF-κB p65 subunit regulates MMP-9 expression [80], while aberrant CpG island methylation disrupts critical pathways involved in disease progression [81].

The oxidative stress-driven pathogenesis of DR involves mitochondrial dysfunction, apoptosis, and autophagy dysregulation, modulated by pathways such as mechanistic target of rapamycin (mTOR)/AMP-activated protein kinase (AMPK) and mediated by VEGF, intercellular adhesion molecule-1 (ICAM-1), NO, and lipid peroxidation. Hyperglycemia generates excess ROS, damaging mitochondrial DNA, promoting the activation of MMP-2 and MMP-9, and facilitating cytochrome c release, leading to cell death [20,66,82,83,84]. Furthermore, NF-κB activation promotes pro-inflammatory and pro-apoptotic responses that intensify cellular damage [85,86,87]. Lipid peroxidation compromises cellular membrane integrity and generates aldehyde byproducts that contribute to neurodegeneration [88,89,90]. These interconnected processes culminate in BRB breakdown, inflammation, and cell death, fueling the progression of DR [19].

## 4. Inflammation and Vascular Alterations

Studies in animal models and diabetic patients have demonstrated that low-grade, chronic inflammation is present throughout multiple stages of DR [91] and represents a pivotal contributor to disease progression, particularly during the early stages [92].

In DR, inflammation is initiated by various stimuli, including hyperglycemia, growth factors, AGEs, circulating cytokines and chemokines, and ROS [93]. These molecular signals are detected by the innate immune system via pattern recognition receptors, such as Toll-like receptors, which recognize pathogen-associated molecular patterns and damage-associated molecular patterns [94]. This activation induces intracellular pathways that promote the expression of pro-inflammatory cytokines (e.g., tumor necrosis factor α (TNF-α), interleukin 1 β (IL-1β), IL-6 and monocyte chemoattractant protein-1 (MCP-1)) [9,21,95].

This pro-inflammatory environment facilitates leukocyte adhesion (leukostasis) to retinal microvasculature, elevates oxidative stress levels, and enhances the expression of adhesion molecules (ICAM-1, VCAM-1), collectively contributing to BRB breakdown, pericyte degeneration, increased vascular permeability, and pathological angiogenesis [96]. Inflammation and angiogenesis exhibit a reciprocal interplay, where each process potentiates the other [97]. ROS, such as hydrogen peroxide (H_2_O_2_) and superoxide anion (${O_{2}^{•}}^{-}$), enhance the production of pro-inflammatory transcription factors like NF-κB, which promotes mediators that upregulate VEGF expression [98]. In turn, VEGF activates and translocates NF-κB to the nucleus, inducing the expression of pro-inflammatory molecules such as ICAM-1, VCAM-1, MCP-1, and cyclooxygenase-2 (COX-2). COX-2, through the synthesis of prostaglandins, stabilizes hypoxia-inducible factor-1 (HIF-1), and further reinforcing VEGF production and NF-κB activation, perpetuating a chronic inflammatory feedback loop [99]. Moreover, VEGF-mediated activation of NF-κB, enhances the production of inflammatory mediators and promotes the NOD-like receptor pyrin domain containing protein 3 (NLRP3) inflammasome activation, leading to IL-1β and IL-18 secretion and contributing to retinal neurodegeneration via pyroptotic mechanisms in Müller glial cells [100].

In parallel, the transcription factor signal transducer and activator of transcription 3 (STAT3) also plays a key role in DR-associated inflammation and angiogenesis. STAT3 activation, induced by pro-inflammatory cytokines and VEGF, promotes endothelial cell proliferation and survival, facilitating neovascularization. Furthermore, STAT3 acts as a central regulator in the amplification of chronic inflammation by inducing the expression of cytokines such as IL-6 and TNF-α, thereby exacerbating vascular dysfunction and increasing BRB permeability. Pharmacological inhibition of STAT3 in rodent models of DR has been shown to attenuate vision loss and reduce retinal damage, underscoring its potential as a therapeutic target [101,102].

DME, a major complication of DR, arises from the compromise of BRB integrity and subsequent accumulation of intra- and extracellular fluid in the macular region [103]. The inner BRB, composed of endothelial cells connected by tight junctions and stabilized by pericytes and glial cells, undergoes alterations induced by hyperglycemia, oxidative stress, and chronic inflammation. The progressive loss of pericytes, which are essential for maintaining capillary integrity, weakens endothelial junctions, increasing vascular permeability and fluid extravasation into the retina. Additionally, the activation of adhesion molecules such as ICAM-1 and leukostasis exacerbates endothelial damage, further disrupting the BRB. As fluid accumulates in the extracellular space, the excessive burden on the retinal pigment epithelium (RPE) impairs its pumping function and homeostatic regulation, compromising the outer BRB. Prolonged inflammation and hypoxic conditions induce VEGF overexpression and dysregulation of RPE ion transport mechanisms, perpetuating fluid accumulation and further compromising BRB function [104,105,106]. This process ultimately leads to the development of DME, the leading cause of vision loss in diabetes, occurring more frequently in advanced stages of the disease [107,108].

## 5. Polyphenols in the Treatment of Diabetic Retinopathy

### 5.1. Oxidative Stress and Antioxidant Defences in DR

The eye is an organ with effective antioxidant defenses that protect it from the oxidative attacks to which it is continuously subjected. The primary source of these attacks lies in the high metabolic activity and oxygen consumption occurring in the retina, as well as in exposure to UV radiation in the anterior portion of the eye. During the physiological activity of the retina, oxidative metabolism in the mitochondria and endoplasmic reticulum produces ROS and reactive nitrogen species [109,110]. The cornea and ocular lenses have the ability to absorb a significant portion of the incident light, thereby protecting the retina from the effects of ultraviolet radiation. However, the production of ROS on the ocular surface can induce molecular changes in deeper structures [111]. Additionally, the production of ROS and reactive nitrogen species can be enhanced by inflammation, exposure to environmental pollutants, genetic factors, aging, or a reduction in antioxidant defenses. Due to their high reactivity, in order to counteract the damage that they can cause to cellular biomolecules with pathophysiological effects, the retina has developed a complex antioxidant defense system that is essential for maintaining ocular homeostasis. However, the imbalance between the production and elimination of these species, which can occur in pathologies such as DM due to exposure to chronic hyperglycemia, triggers diseases such as DR.

The cellular antioxidant defense system is typically classified into two major groups: the enzymatic system, primarily composed of superoxide dismutase (SOD), catalase (CAT), GSH peroxidase, and heme oxygenase, and the non-enzymatic antioxidant system, consisting of molecules capable of reducing or chelating oxidizing species such as GSH [112]. Furthermore, exogenous antioxidants such as vitamins (C, E), carotenoids (lutein, zeaxanthin), flavonoids, coenzyme Q10, and alpha-lipoic acid may also participate in retinal protection mechanisms [113].

${O_{2}^{•}}^{-}$ is one of the major reactive species involved in retinal damage. It has been shown that its levels increase in cell culture experiments at high glucose concentrations and in retinal tissue in various animal models of diabetes compared to healthy animals [114,115,116,117]. In the diabetic retina, ${O_{2}^{•}}^{-}$ is primarily generated at the mitochondrial level and through the activity of NADPH oxidase (Nox) [118]. The removing of this free radical is catalyzed by SOD, which transforms ${O_{2}^{•}}^{-}$ to a less reactive molecule, hydrogen peroxide (H_2_O_2_) [119]. The action of peroxidases and CAT avoid the oxidative damage caused by the accumulation of H_2_O_2_. In different DR animal models, the modulation of these ROS has been related with retinal damage reduction. In fact, a reduction in SOD and CAT activity has been observed in the diabetic rat and rabbit retinas [117,120]. Overexpression of SOD2 protected retinas of diabetic mice against the development of acellular retinal capillaries [121]. Therefore, although chronic exposure to glucose is a potent inducer of ROS capable of overcoming the antioxidant defenses of the retina, interventions that stimulate the endogenous antioxidant capability may be a promising therapeutic approximation to avoid the development of DR. However, to ensure the success of this kind of therapeutic approach, it seems critical both start the interventions as soon as possible, before irreparable damage has occurred and phenotypic consequences, such as angiogenesis, are observable, and to sustain the intervention over time.

### 5.2. Polyphenol Supplements Against DR

Between the late 20th century and the early 21st century, scientific interest in the beneficial effects of natural antioxidants has steadily increased. Since the proposal of the French paradox and the attribution of the cardiovascular protective effects of resveratrol found in wine [122], numerous studies, both in vitro and in vivo, have been conducted, delving into the antioxidant properties of a wide range of phytochemicals, such as polyphenols, which are present in the diet. In parallel, a variety of techniques have been developed to assess antioxidant capacity [123] and its implications in pathologies associated with increased oxidative stress. For instance, polyphenols such as resveratrol, curcumin, pterostilbene, quercetin, and epigallocatechin gallate have shown beneficial effects in diseases characterized by oxidative imbalance, including cancer [124,125,126,127,128], diabetes [129,130,131,132,133], and Alzheimer’s disease [134,135,136,137,138].

In the development of DR, as previously discussed, oxidative stress induced by chronic hyperglycemia is a key contributor. The imbalance between antioxidant defenses and oxidative insults triggers subclinical damage that progressively worsens, such as ganglion cell neurotoxicity and pericyte cell death. These early effects, which are etiologically linked to ROS, do not result from a single molecular pathway but rather from a broad cascade of reactions that exacerbate retinal damage. As there is no unique disrupted pathway, and unless the root cause, diabetes, is cured, treatments targeting only one molecular mechanism are unlikely to be effective. A large body of literature highlights the complex protective role of polyphenols in DR, acting on multiple molecular targets. This section provides a comprehensive review of the principal polyphenols and the molecular mechanisms by which they confer therapeutic benefits in DR.

#### 5.2.1. Resveratrol

Among the numerous studies conducted on animal models, resveratrol has been shown to mitigate the diabetes-induced downregulation of occludin and the upregulation of pro-inflammatory HMGB1 and RAGE in the retinas of diabetic rats, as well as the degradation of the BRB. These findings are particularly relevant, as the same study also demonstrated increased HMGB1 expression in the vitreous humor of patients with PDR [84]. Resveratrol’s anti-inflammatory, antioxidant, and anti-apoptotic activities in experimental DR animal models include: promoting AMPK activity [139], inhibiting SIRT1 deactivation [84,139], suppressing NF-κB phosphorylation [139], preventing apoptosis in retinal neurons [140], Müller glial cells [141], RPE cells [142], protecting against ferroptosis regulating the Nrf2/glutathione peroxidase 4 (GPx4)/PTGS2 pathway [143], and reducing inflammatory factors such as IL-1β, IL-6, TNF-α, VEGF, Interferon-γ, MCP-1 [144], COX-2 [145], and TGF-β1, IL8 [146], along with the GSSG/GSH ratio and lipid peroxidation index [147].

#### 5.2.2. Piceatannol

Although experiments were not conducted under high-glucose conditions, piceatannol, a natural stilbene derived from resveratrol, has been shown to enhance antioxidant defenses and prevent H_2_O_2_- induced cell death in ARPE-19 cells through the activation of the phosphoinositide 3-kinase (PI3K)/Akt–Nrf2/HO-1 signaling pathway [148].

#### 5.2.3. Quercetin

Despite substantial preclinical evidence, the clinical use of quercetin in DR remains limited, mainly due to its poor bioavailability, the lack of suitable ocular formulations, and rapid degradation [149]. In human retinal microvascular endothelial cells, quercetin reduces cell migration and tube formation, as well as the expression of NLRP3, apoptosis-associated speck-like protein (ASC), caspase-1, IL-1β, IL-18, microtubule-associated proteins 1A/1B light chain 3B (LC3-B), Beclin-1, and autophagy under high-glucose conditions, in a dose-dependent manner [150].

In diabetic rat models, intraperitoneal administration of quercetin decreases cell apoptosis, improves retinal histopathology, increases the number of ganglion cells, and suppresses the overexpression of pro-inflammatory cytokines (IL-1β, IL-18, IL-6, TNF-α), HMGB-1, and NLRP3, as well as proangiogenic factors such as VEGF, ICAM-1 [151], and MMP9 [152]. Simultaneously, it promotes the expression of neurotrophic factors, including brain-derived neurotrophic factor and nerve growth factor, likely through the induction of heme oxygenase-1 (HO-1) [151].

#### 5.2.4. Anthocyanins

Anthocyanins, a class of flavonoids found in fruits such as blueberries, blackberries, and black grapes, have demonstrated notable protective effects in DR. These compounds reduce the production of ROS and malondialdehyde (MDA), a key oxidative stress and lipid peroxidation marker, in diabetic rat retinas, and lower VEGF and IL-1β levels in serum. Concurrently, they increase GSH content and the expression of the antioxidant enzyme GPx, accompanied by an upregulation and nuclear translocation of Nrf2 and HO-1 [153].

In vivo studies have shown that blueberry-derived anthocyanins mitigate diabetes-associated outcomes such as weight loss, hyperglycemia, and retinal cell apoptosis. Moreover, in diabetic mice, they reduce plasma IL-1β levels and attenuate the inflammatory response in a dose-dependent manner, improve retinal architecture, and prevent early retinal damage. These effects are associated with enhanced phosphorylation of AKT and glycogen synthase kinase 3 beta, stimulation of glucagon-like peptide-1 release, and increased retinal expression of the glucagon-like peptide-1 receptor, suggesting a role in restoring insulin signaling and offering neuroprotective and anti-inflammatory benefits [154].

#### 5.2.5. Epigallocatechin Gallate

Epigallocatechin gallate, the most abundant and bioactive polyphenol in green tea, has shown beneficial effects in diabetic rat retinas by restoring GSH levels, enhancing the activity of antioxidant enzymes such as SOD and CAT, and reducing TNF-α, VEGF, and capillary basement membrane thickening [155]. Beyond its antioxidant action, epigallocatechin gallate prevents the overexpression of glial fibrillary acidic protein (GFAP) and the loss of occludin, while positively modulating neuronal NOS and the AKT/endothelial NOS pathway, contributing to the preservation of BRB integrity by limiting VEGF-induced permeability [156].

In vitro, epigallocatechin gallate inhibits ARPE-19 cell migration and adhesion by blocking PI3K/Akt, MEK/extracellular signal-regulated kinase 1/2 (ERK1/2), and p38 mitogen-activated protein kinase (MAPK) pathways [157]. Additionally, in high-glucose-treated Müller cells, EGCG promotes autophagy by inhibiting the mTOR pathway, sustaining LC3-B and Beclin-1 levels, and decreasing p62 expression, thereby facilitating autophagosome and autolysosome formation [158].

#### 5.2.6. Naringenin

Naringenin, a citrus-derived flavonoid, exhibits both antioxidant and anti-inflammatory properties in experimental models of DR. In streptozotocin-induced diabetic rats, naringenin improves redox balance, as evidenced by decreased lipid peroxidation [159] and increased levels of GSH, SOD, and CAT [159,160]. It downregulates the expression of NF-κB and pro-inflammatory cytokines such as TNF-α, IL-1β, and IL-6 [160].

In the diabetic retina, naringenin enhances the expression of neuroprotective markers, including synaptophysin and brain-derived neurotrophic factor, along with its receptor tropomyosin receptor kinase B, and exerts antiapoptotic effects, contributing to retinal protection [159]. In treated diabetic rats, it also improves body weight in a dose-dependent manner, reduces serum glucose levels, increases ganglion cell layer thickness and cell number, and lowers GFAP expression [160].

#### 5.2.7. Eriodictyol

Eriodictyol, a polyphenol found in various fruits and vegetables, has demonstrated notable anti-inflammatory and antioxidant effects [161,162]. In diabetic rat retinas, eriodictyol treatment significantly reduces TNF-α, ICAM-1, VEGF, and endothelial NOS levels in a dose-dependent manner. It also lowers retinal lipid peroxidation and mitigates BRB breakdown [163].

Lv et al. further explored its role in DR through in vitro experiments on ganglion cells. Eriodictyol treatment under high-glucose conditions decreased apoptosis and reduced TNF-α and IL-8 production. Additionally, it enhanced the expression of antioxidant enzymes such as SOD, GPx, and CAT, thereby strengthening the retinal antioxidant defense system. These effects were associated with the activation of the Nrf2/HO-1 pathway [164].

#### 5.2.8. Myrecetin/Galic Acid

Myricetin is a polyphenol with antioxidant and anti-inflammatory properties, present in vegetables, fruits, nuts, berries, tea, and red wine. Although in vivo evidence is limited, several in vitro studies support its potential in DR. In ARPE-19 cells exposed to oxidative stress, myricetin promotes cell survival by activating SOD and Nrf2, while downregulating inducible NOS at the transcriptional level [165]. In retinal pericytes, myricetin inhibits AGE-induced cell migration by modulating the RAGE-Src-ERK1/2-FAK-paxillin signaling cascade, a key process in microvascular destabilization [166].

In diabetic rats, co-treatment with myricetin and gallic acid lowers hyperglycemia, improves histopathological retinal features, suppresses TNF-α and prostaglandin-E2 overexpression, reduces vascular leakage and neovascularization, and lowers the MDA/GSH ratio [167].

#### 5.2.9. Scutellarin

Scutellarin, a flavone extracted from *Scutellaria baicalensis*, has demonstrated promising effects in DR. It significantly increases the activity of GPx, SOD, and CAT, reduces retinal inflammation, and ameliorates histopathological alterations induced by chronic hyperglycemia in diabetic rats, potentially through inhibition of NLRP3 inflammasome activation [168]. Furthermore, scutellarin protects retinal ganglion cells from pyroptosis by inhibiting caspase-1, gasdermin D, NLRP3, IL-1β, and IL-18 [169].

In addition, scutellarin reduces proliferation, migration, and tube formation in human retinal endothelial cells. In vivo, it attenuates microvascular neovascularization in type II diabetic rats by suppressing VEGF, p-ERK, p-FAK, and p-Src expression [170]. In diabetic mouse models, it also protects against oxidative stress-induced BRB damage by reducing claudin-1 and claudin-19 expression, downregulating microglial activation, inhibiting NF-κB and TNF-α expression, and suppressing p-ERK signaling both in vivo and in vitro [171].

#### 5.2.10. Rhaponticin

Rhaponticin, a natural stilbenoid glucoside compound, exhibits potent antioxidant activity in vivo by activating the Nrf2/HO-1/NF-κB signaling pathway, thereby enhancing the retinal expression of key antioxidant enzymes, including HO-1, SOD, CAT, GPx, and NAD(P)H quinone dehydrogenase 1. Additionally, rhaponticin reduces levels of MDA, TNF-α, and MMP-2, while increasing IL-10 and tissue inhibitor of metalloproteinases-1, indicating a favorable shift toward an anti-inflammatory and redox-balanced retinal microenvironment [172].

#### 5.2.11. Curcumin

Curcumin enhances the expression of the antioxidant transcription factor Nrf2 [173], reduces retinal lipid peroxidation [174,175], increases SOD levels, alleviates oxidative stress [175], decreases VEGF expression, reduces basement membrane thickness of retinal capillaries [176], and lessens vascular tortuosity and microaneurysm formation [177].

For instance, administration of piperine/curcumin (1 g/day) for 12 weeks to 30 NPDR patients increased their total antioxidant capacity and SOD activity while reducing lipid peroxidation, as assessed by MDA levels. However, no macroscopic ocular improvements were observed via optical coherence tomography (OCT) or OCTA compared to 30 placebo-treated patients [178].

#### 5.2.12. Ferulic Acid/Chlorogenic Acid/Arctiin/Rutin

In both in vitro and in vivo experimental models, the He-Ying-Qing-Re formula, whose main active constituent is ferulic acid, has demonstrated beneficial effects on DR by modulating multiple pathophysiological pathways [179]. In human umbilical vein endothelial cells, ferulic acid inhibits the formation of AGEs and attenuates the associated inflammatory response by suppressing the activation of the NF-κB and p38 MAPK signaling pathways [180]. Furthermore, the combination of ferulic acid with other polyphenols such as chlorogenic acid and arctiin prevents degeneration of the retinal vasculature and preserves the integrity of the BRB. This is achieved by maintaining the expression of tight junction proteins, including claudin-1 and ZO-1, without significantly affecting hyperglycemia. In addition, this polyphenolic combination suppresses activation of the AGEs receptor and its downstream signaling via Akt [181].

The combination of ferulic acid, chlorogenic acid, and rutin has also been shown to attenuate retinal ganglion cell loss and retinal thinning in diabetic mice. Moreover, it inhibits both endoplasmic reticulum stress and mitochondrial oxidative stress in retinal ganglion cells in vitro by downregulating ATF4-dependent pathways, thereby reducing apoptosis [182]. Notably, prolonged treatment with ferulic acid in a diabetic murine model (C57BL/KsJ db/db) protects against retinal structural deterioration, preserving critical layers such as the ganglion cell layer, inner nuclear layer, and inner plexiform layer [183].

#### 5.2.13. Pterostilbene

Our group demonstrated that pterostilbene, via NRF2, one of the key regulators of redox homeostasis, can prevent ganglion cell damage in the early stages of DR without affecting glycemia or body weight in experimental animals. Polyphenol treatment restored the reduced activities of SOD, CAT, and GPx observed in the retinas of diabetic rabbits, alongside a decrease in H_2_O_2_ levels. Furthermore, the GSH/GSSG ratio, which was reduced in diabetic retinas, was normalized after pterostilbene treatment [117]. Decreased GSH levels have also been reported in other experimental models, such as diabetic rat retinas [184] or pericyte cultures exposed to high glucose levels [185]. Additionally, reduced levels of 4-HNE were observed [117], and modulation of the antioxidant machinery by pterostilbene supplementation prevented early neuroretinal damage and lipid peroxidation of the most abundant retinal fatty acids: docosahexaenoic acid (DHA; 22:6n-3), arachidonic acid (20:4n-6), and adrenic acid (22:4n-6) [186,187,188]. These fatty acids play a physiological role in protecting against the development of various retinal disorders, including DR [189]. The increase in products derived from their lipid peroxidation has been associated with neuronal damage [190] and the development of neovascularization in DR [191,192].

Spectrometry analyses have shown that lipid peroxidation in the retinas of diabetic rabbits increases the levels of: PGE_2_, PGF_2_α, isoprostanes (8-iso-PGE_2_, 8-iso-15-keto-PGF_2_α, and 8-iso-15-(R)-PGF_2_α), 17-F2t-dihomo-IsoP, Ent-7(RS)-7-F2t-dihomo-IsoP, 17-epi-17-F2t-dihomo-IsoP, 17(RS)-10-epi-SC-∆^15^-11-dihomo-IsoF, 10-epi-10-F4t-NeuroP, 4(RS)-4-F4t-NeuroP, and 14(RS)-14-F4t-NeuroP. Pterostilbene treatment was able to reduce the levels of these lipid peroxidation products in diabetic rabbits to values similar to those in controls [193].

As previously mentioned, current therapeutic approaches against DR are typically implemented when irreversible damage has already occurred, such as induced neurotoxicity or abnormal blood vessel formation. Targeting a specific molecule like VEGF or photocoagulating blood vessels are palliative strategies aimed at temporarily halting the progression of irreversible retinal damage. For this reason, the pleiotropic effects [194] of polyphenols may underlie the clinical benefits of their use, benefits that go beyond a singular, directed, and dominant primary effect. However, to increase the likelihood of success, early intervention is crucial. Thus, in order to prevent DR development, these compounds should be administered at the time of diabetes diagnosis. Both we and other researchers, for example [113], think that although human studies are limited; this hypothesis is supported by numerous in vitro and in vivo investigations and by the beneficial outcomes observed in patients with DR following polyphenol supplementation.

Recently, our group demonstrated in a pilot clinical study that pterostilbene treatment for one year improved systemic redox status and reduced the progression of retinal vascular alterations. Moreover, after a six-month treatment interruption, both the redox imbalance and retinal damage indicators reappeared [195]. These studies are temporally limited and therefore do not capture the evident macroscopic progression of the disease due to its slow development in patients with good glycemic control. Nevertheless, the results suggest that polyphenol treatment may further delay disease progression, offering clear advantages for patients.

### 5.3. Nanotechnology for Polyphenol Delivery

Among the evident limitations of polyphenol-based treatments are their low bioavailability, difficulty in reaching biologically effective concentrations, and poor localization in target organs. Conventional therapies against DR, as previously discussed, also present several limitations and side effects. The innovative development of nanomedicine is enabling new strategies to overcome these challenges. In recent years, new materials with evident advantages for the treatment of DR have been developed. Among their key characteristics are small particle size, high penetration through the BRB, good biocompatibility, the ability to reduce the elimination rate of therapeutics by allowing sustained release, and favorable physicochemical properties of the nanoparticles [196].

Although the progress and clinical validation of this approach still require further efforts, the use of nanomaterials as carriers enables controlled, localized, and sustained delivery of therapeutic agents to the retinal tissue. This reduces the side effects associated with conventional treatments and represents a significant advancement toward personalized and precision medicine for DR [196,197,198]. Recently, Baghban et al. published an excellent review presenting numerous studies that employ nanotechnology to improve the diagnosis and treatment of DR [196].

In the treatment of DR at more advanced stages, nanoparticles carrying various types of anti-angiogenic molecules have shown promising results. For example, in an effort to reduce the frequency of intraocular anti-VEGF injections, Du Toit et al. designed a nanosystem for the controlled release of the anti-angiogenic peptide p11. The nanoparticles developed enabled sustained release of the peptide for up to 60 days, with a loading efficiency of 67% and biocompatibility ranging from 97% to 99% [199]. Similarly, encouraging results have been obtained by encapsulating other anti-angiogenic drugs into various types of nanoparticles, such as Apatinib, a VEGFR-2 inhibitor [200,201]; Bevacizumab, a monoclonal antibody against VEGF [202]; and nanoparticles loaded with small interfering RNA targeting the human antigen R (HuR), an RNA-binding protein that stabilizes VEGF mRNA and promotes its expression, an essential factor in the progression of DR [203]. Additionally, lipid-encapsulated natural compounds such as naringenin, a polyphenol abundant in citrus fruits, have demonstrated anti-angiogenic effects in animal models of retinopathy comparable to those of bevacizumab [204] (Table 2). Another example of liposomes encapsulation is ellagic acid, a polyphenol capable of downregulating VEGF, GFAP, HIF-1α, and VEGFR-2 expression in murine models of DR [205] (Table 2).

Nevertheless, despite fewer studies in this area, it may be of greater interest to focus on protecting the diabetic retina from oxidative stress and inflammation, which are the underlying causes of vascular alterations observed during disease progression. Treatment with nanoparticles co-loaded with curcumin and insulin halted disease progression, stabilized blood glucose levels, improved liver function, reduced AGEs, enhanced ATP synthesis, and protected the retina from vascular damage induced by hyperglycemia. Moreover, this therapy did not exhibit any nanoparticle-induced toxicity in experimental animals [206]. In addition, new advanced encapsulation strategies for curcumin have been explored, such as proniosomal gels, transferosomes, and cyclodextrin complexes, which have enhanced its stability and therapeutic efficacy in deep ocular tissues, strengthening its antioxidant action in DR [207] (Table 2).

Nanoparticles encapsulating pyrrolidine dithiocarbamate and triamcinolone acetonide demonstrated antioxidant activity in a diabetic animal model by reducing protein carbonylation and lipid peroxidation, and they protected against the development of vascular damage and retinal neovascularization [208].

The encapsulation of diosmin (3’,5,7-trihydroxy-4’-methoxyflavone-7-rhamnoglucoside), a natural flavone glycoside derived from hesperidin, a flavanone glycoside abundant in citrus fruits [209], in nanostructured lipid carriers enhanced its water solubility for potential ophthalmic topical application. Studies in RPE cells confirmed its antioxidant and anti-inflammatory properties, making it a potential candidate for DR treatment [210] (Table 2).

Treatment with gold nanoparticles coated with resveratrol in diabetic rats improved various retinal parameters affected by hyperglycemia. For instance, it reduced disease-induced retinal vascular permeability, increased the thickness of the inner nuclear layer and ganglion cell layer, and decreased the expression of VEGF, TNF-α, IL-6, IL-1β, NF-κB, vascular cell adhesion molecule-1, ICAM-1, and MCP-1 [211] (Table 2).

Other natural compounds, such as green tea catechins, particularly epigallocatechin gallate, have been nanoformulated into cationic lipid-based systems, significantly enhancing ocular permeability and bioavailability [212], whereas several phenolic acids, including gallic, ferulic, caffeic, chlorogenic, and rosmarinic acids, have been encapsulated using platforms such as lipid nanoparticles, silica nanoparticles, gold nanoparticles, poly (lactic-co-glycolic acid), and chitosan-based carriers. These nanocarriers have demonstrated the capacity to modulate key neuroinflammatory and oxidative stress-related signaling pathways, including NF-κB, MMP-9, and VEGF, thereby exhibiting promising therapeutic potential for the prevention and management of neurodegenerative processes associated with DR and other oxidative stress-related pathologies [213].

An additional innovative strategy involves the use of ROS-responsive nanoparticles, such as those loaded with essential oils derived from fructus *Alpiniae zerumbet*. These nanocarriers have shown efficacy in attenuating Müller cell activation and mitigating oxidative stress and inflammation during the early stages of DR [214].

Moreover, fenofibrate encapsulated within biodegradable poly (lactic-co-glycolic acid) nanoparticles has been shown to effectively reduce retinal vascular leakage, inhibit retinal leukostasis, and downregulate the overexpression of VEGF and ICAM-1 in experimental DR models, thereby enhancing retinal function without inducing detectable toxicity [215].

Furthermore, myricetin, a polyphenolic flavonoid with potent antioxidant properties, has recently been incorporated into a thermosensitive in situ, forming nanoemulgel to optimize ocular delivery. This formulation exhibited markedly enhanced corneal permeation and retention, favorable ocular biocompatibility, and a significant antioxidant and antiproliferative effect comparable to bevacizumab in retinoblastoma cell lines, positioning it as a promising non-invasive therapeutic candidate for the treatment of DR [216] (Table 2).

Antioxidant strategies based on nanotechnology have also expanded to include other natural flavonoids such as rutin and quercetin. Treatment with phyto-reduced nanoparticles loaded with rutin in an early-stage DR and cataract animal model showed superior antioxidant capacity compared to treatment with insulin and rutin, as indicated by analyses of MDA, CAT, and SOD levels [217] (Table 2). Likewise, the combination of quercetin with low-toxicity iron ions has emerged as a promising treatment, capable of mimicking antioxidant enzyme activity. Beyond their action against ROS, these compounds exhibit multitarget effects that confer both anti-inflammatory and anti-angiogenic properties relevant to DR [218].

**Table 2:** Polyphenols and nanotechnology: Mechanisms, therapeutic effects, and limitations.

Polyphenol	Antioxidant Mechanism	Anti-Inflammatory Pathways Modulated	Comparative Effect vs. Conventional Therapies	Nanoparticle Type and Effect on Bioavailability	Limitations/Future Directions	Refs
Naringenin	VEGF inhibition, anti-angiogenic activity	Potential indirect inhibition of inflammatory mediators	Comparable efficacy to bevacizumab in animal models	Liposomal/solid lipid carriers; improved retinal penetration	Requires clinical validation; limited in vivo studies	[204]
Ellagic acid	Downregulation of VEGF, HIF-1α, GFAP	Potential neuroprotective anti-inflammatory effects	Promising multitarget anti-angiogenic activity	Liposomes; adequate tissue retention	Few direct comparisons; limited in vivo evidence	[205]
Curcumin	Reduces AGEs, enhances ATP synthesis	Decreases oxidative stress and pro-inflammatory cytokines	Complementary to insulin; systemic metabolic effects	Co-encapsulated in nanogels, liposomes, cyclodextrins	Low stability; lacks extensive clinical trials	[206,207]
Resveratrol	ROS scavenging, lipid peroxidation inhibition	NF-κB, IL-6, IL-1β, TNF-α, VCAM-1, ICAM-1	Multitarget effects; comparable to anti-VEGF in some aspects	Gold nanoparticles; markedly enhances ocular bioavailability	Formulation standardization needed	[211]
Diosmin	Antioxidant activity in RPE cells	Reduces pro-inflammatory mediators	Potential topical ocular treatment	Nanostructured lipid carriers; improves aqueous solubility	Preclinical validation still lacking	[210]
Myricetin	Sustained antioxidant ROS reduction	Antiproliferative and ocular anti-inflammatory effects	Comparable to bevacizumab in cell models	Thermosensitive nanoemulgel; high corneal retention and biocompatibility	Lacks in vivo human data	[216]
Rutin	Increases SOD, CAT; lowers MDA	Indirect antioxidant activation with anti-inflammatory potential	Greater antioxidant effect than insulin-rutin combination	Phyto-reduced nanoparticles; enhanced delivery	Preliminary studies; not compared to standard therapies	[217]
Quercetin	Enzyme-like antioxidant capacity	NF-κB, IL-6, VEGF (indirectly)	Combined anti-inflammatory and anti-angiogenic action	Complexed with low-toxicity iron; sustained release	Early-stage mechanistic research	[218]

AGE(s): Advanced glycation end products; ATP: Adenosine triphosphate; CAT: Catalase; GFAP: Glial fibrillary acidic protein; HIF-1α: Hypoxia-inducible factor 1-alpha; ICAM-1: Intercellular adhesion molecule-1; IL: Interleukin; MDA: Malondialdehyde; NF-κB: Nuclear factor kappa B; ROS: Reactive oxygen species; RPE: Retinal pigment epithelial; SOD: Superoxide dismutase; TNF-α: Tumor necrosis factor Alpha; VCAM-1: Vascular adhesion molecule-1; VEGF: Vascular endothelial growth factor.

## 6. Relevance of Polyphenol Therapy Against DR

Although, as shown, therapeutic approaches for advanced stages of DR have been developed in recent years, the reality is that not all diabetic patients suffering from this complication have access to such treatments. Low-income countries, regions, or individuals often face barriers to therapies and specific equipment that are, in many cases, simply inaccessible. Several studies have examined the relationship between socioeconomic status and diabetes-related microvascular complications.

In the United Kingdom, a prospective study including 28,339 patients from the UK Biobank demonstrated that individuals with higher income levels and lower Townsend deprivation index scores had a lower risk of developing DR [219]. Similarly, associations between area-level socioeconomic status and the development, progression, and vision loss due to DR have been reported in Asian [220] and Canadian populations [221]. The relationship between ocular diabetes-related complications and socioeconomic status can be attributed to several factors including:

- Dietary patterns linked to socioeconomic status. A pro-oxidative diet, rich in red meat, sugar, processed foods, and saturated and trans fats, and low in fruits, vegetables, and mono- and polyunsaturated fats, has been associated with the development of diabetes [222] and DR [223]. Furthermore, this association is reinforced by the fact that healthier eating habits, such as diets rich in polyphenols, are more common among people with a higher socioeconomic status [224].
- Lack of trust in healthcare systems among low-income individuals, which may lead to lower engagement with healthcare services [224].
- Limited capacity to attend regular screenings, either due to the geographical distance of disadvantaged neighborhoods from ophthalmology clinics [225] or due to transportation difficulties or securing time off from work [226].
- Low health literacy, often tied to lower educational attainment and socioeconomic status. Understanding and appropriately applying health information is crucial for enabling individuals to make informed decisions and actively participate in disease prevention and treatment [227]. In this context, poor glycemic control, often associated with lack of awareness and financial limitations, has serious implications for the development of DR [228].
- Inability to afford healthcare costs, especially when access to vision care is dependent on insurance coverage, further limits access to treatment for individuals with fewer economic resources [228,229].

The Mediterranean diet has emerged as a promising non-pharmacological intervention for the management of diabetes and DR, as it can improve glycemic control, reduce oxidative stress and inflammation, and slow the progression of DR [230]. Among its components, polyphenols stand out as the most abundant phytochemicals, with substantial evidence supporting their benefits in chronic diseases such as diabetes [231]. As previously discussed, their antioxidant and anti-inflammatory properties offer protective effects against the development and progression of DR.

Despite limitations in their bioavailability, the direct use of polyphenols or their integration into delivery systems may help address many of the challenges associated with conventional therapies against DR. First, such approaches could help avoid the adverse effects linked to some current treatments. Furthermore, due to their lower cost, they could be used by the most vulnerable population and in impoverished regions. Some polyphenols have shown potential in supporting glycemic control, a key factor in preventing diabetes-related complications. Administering them from the moment of diabetes diagnosis could help to delay or prevent the onset and progression of DR and other chronic hyperglycemia-related complications, thereby significantly reducing the economic burden on national healthcare systems. When combined with effective patient education and communication strategies, polyphenol-based interventions may also help address antioxidant deficiencies and the high cost of care among socioeconomically disadvantaged populations. Moreover, slowing disease progression could allow for longer intervals between regular screening exams, making disease monitoring more feasible and accessible.

## 7. Conclusions

DR is a microvascular complication resulting from chronic hyperglycemia in the context of DM. Despite notable advances in diagnostic techniques over recent years, DR is commonly diagnosed upon the appearance of clinically visible lesions, such as microaneurysms, hard exudates, and retinal hemorrhages, hallmarks of retinal vascular damage. Consequently, current therapeutic interventions primarily aim to limit the progression of microvascular alterations to prevent irreversible vision loss.

Recent developments in imaging technologies, particularly OCTA, have enabled the acquisition of high-resolution, wide-field structural images of the retina. These advancements not only allow for improved visualization of peripheral retinal areas but also facilitate quantitative assessment of retinal thickness. OCT studies have revealed a significant thinning of the inner retinal layers, specifically involving the ganglion cell layer and the retinal nerve fiber layer [232,233,234,235]. This progressive thinning, and, consequently, the loss of retinal neurons, precedes the onset of microvascular lesions [232,234], leading to the redefinition of DR as a neurovascular complication [236]. While OCTA has already demonstrated significant utility in the diagnosis of DR, its full potential has yet to be realized. The in-depth microvascular analysis capabilities of OCTA, such as vascular density measurement, fractal dimension, tortuosity, and expanded field imaging, combined with longitudinal clinical studies and the integration of artificial intelligence capable of correlating imaging data with biochemical and molecular findings, are poised to become essential tools for both the diagnosis and prognosis of DR.

Currently, there is no treatment available that can cure DR. As previously noted, existing therapeutic strategies target vascular abnormalities that appear once irreversible cellular and molecular retinal damage has occurred. Although scientific advancements have extended the lifespan of individuals with diabetes by transforming the disease into a manageable chronic condition, this increased longevity is frequently accompanied by complications such as DR. In this context, it is proposed that, in addition to strict glycemic control, early intervention strategies should be implemented, ideally prior to the onset of phenotypically detectable signs of retinal damage, and as early as the initial diagnosis of diabetes.

Given the strong association between chronic hyperglycemia, oxidative stress, and the development of DR, antioxidant therapies may offer significant benefits. Although there are a limited number of long-term clinical studies involving large patient cohorts, polyphenols have emerged as a promising therapeutic approach. Regardless of whether they are used individually or in combination, polyphenols have demonstrated efficacy at the cellular level in slowing disease progression to advanced stages. However, a major limitation of such therapeutic strategies lies in their low bioavailability and poor tissue targeting. These challenges may be overcome through the development of novel delivery systems, such as those enabled by nanotechnology.

## Figures and Tables

**Table 1:** Available intravitreal drugs for diabetic macular edema.

Intravitreal Drug	Year of Approval by the FDA/EMA	Drug Type	Approximated Cost per Injection (USA Dollars)	Biosimilars for Anti-VEGF (Approval Date and Agency)
Bevacizumab 1.25 mg (Avastin^®^)	2004/2005 (off-label for ocular pathologies)	Humanized monoclonal antibody (anti-VEGF-A)	50 $	Mvasi (2017, FDA) Zirabev (2019, FDA)
Ranibizumab 0.3 or 0.5 mg (Lucentis^®^)	2006/2007	Humanized monoclonal antibody (anti-VEGF-A)	1950 $	Byooviz (2021 FDA/EMA) Cimerli (2022, FDA) Ranivisio (2022 EMA) Ximluci (2022 EMA) Epruvi (2024 EMA)
Dexamethasone implant 0.7 mg (Ozurdex^®^)	2010/2010	Synthetic steroid	1500 $	
Aflibercept 2 mg (Eylea^®^ 2 mg)	2011/2012	Fusion protein (soluble VEGF receptor)	1850–2000 $	Yesafili (2024 FDA/2023 EMA) Opuviz (2024 FDA/EMA) Pavblu (2024 FDA/2025 EMA) Ahzantive (2024 FDA/2025 EMA) Baiama (2025 EMA) Eydenzelt (2025 EMA) Afqlir (2024 EMA) Vgenfli (Authorization pending EMA)
Fluocinolone Acetonide 0.19 mg implant (Iluvien^®^)	2014/2019	Synthetic steroid	6000–7000 $	
Brolucizumab 6.0 mg (Beovu^®^)	2019/2020	Humanized monoclonal antibody (anti-VEGF-A)	1850–2000 $	Not available
Faricimab 6.0 mg (Vabysmo^®^)	2022/2022	Humanized bispecific monoclonal antibody (anti-VEGF-A and anti-Ang-2)	2289 $	Not available
Aflibercept 8 mg (Eylea^®^ HD)	2023/2014	Fusion protein (soluble VEGF receptor)	3000–4000 $	

Ang-2: Angiopoietin-2; EMA: European Medicines Agency; FDA: Food and Drugs Administration; HD: High dose; VEGF: Vascular endothelial growth factor. FDA-approved biosimilars for anti-VEGF therapies can be found at: https://www.fda.gov/drugs/biosimilars/biosimilar-product-information, (accessed on 12 July 2025), EMA-approved biosimilars for anti-VEGF therapies are available at: https://www.ema.europa.eu/en/medicines, (accessed on 12 July 2025).

## Abbreviations

AGE(s): Advanced glycation end products; AKT: Protein Kinase B; AMPK: AMP-activated protein kinase; Ang-2: Angiopoietin-2; ATP: Adenosine triphosphate; BRB: Blood-retinal barrier; CAT: Catalase; CHOP: C/EBP homologous protein; COX-2: Cyclooxygenase-2; DME: Diabetic macular edema;DM: Diabetes mellitus;DM1: Diabetes mellitus type 1; DM2: Diabetes mellitus type 2; DR: Diabetic retinopathy; ERK: Extracellular signal-regulated kinase; FDA: Food and Drug Administration; GFAP: Glial fibrillary acidic protein; GSH: Glutathione (reduced form); GSSG: Glutathione disulfide (oxidized form); H_2_O_2_: Hydrogen peroxide; HIF-1α: Hypoxia-inducible factor 1-alpha; HO-1: Heme oxygenase-1; ICAM-1: Intercellular adhesion molecule-1; ICDRSS: International Classification of Diabetic Retinopathy Severity Scale; IL: Interleukin; LC3-B: Microtubule-associated proteins 1A/1B light chain 3B; MAPK: Mitogen-activated protein kinase; MCP-1: Monocyte chemoattractant protein-1; MDA: Malondialdehyde; MMP: Matrix metalloproteinase; mTOR: Mechanistic target of rapamycin; NAD^+^: Nicotinamide adenine dinucleotide; NADPH: Nicotinamide adenine dinucleotide phosphate; NF-κB: Nuclear factor kappa B; NLRP3: NOD-like receptor pyrin domain containing protein 3; NOS: Nitric oxide synthase; NPDR: Non-proliferative diabetic retinopathy; NRF2/Nrf2: Nuclear factor erythroid 2–related factor 2; OCT: Optical coherence tomography; OCTA: Optical coherence tomography angiography; ${O_{2}^{•}}^{-}$: Superoxide anion; PDR: Proliferative diabetic retinopathy; PI3K: Phosphoinositide 3-kinase; PKC: Protein kinase C; PRP: Panretinal photocoagulation; RAGE: Receptor for advanced glycation end products; ROS: Reactive oxygen species; SOD: Superoxide dismutase; STAT3: Signal transducer and activator of transcription 3; TGF-β: Transforming growth factor beta; TNF-α: Tumor necrosis factor Alpha; VA: Visual acuity; VEGF: Vascular endothelial growth factor.
